# Tea polyphenols inhibit the activation of NF-κB and the secretion of cytokines and matrix metalloproteinases by macrophages stimulated with *Fusobacterium nucleatum*

**DOI:** 10.1038/srep34520

**Published:** 2016-10-03

**Authors:** Amel Ben Lagha, Daniel Grenier

**Affiliations:** 1 Oral Ecology Research Group, Faculty of Dentistry, Université Laval, Quebec City, QC, Canada

## Abstract

*Fusobacterium nucleatum* has been associated with both periodontal disease and inflammatory bowel disease. This Gram-negative bacterium possesses a high inflammatory potential that may contribute to the disease process. We hypothesized that green and black tea polyphenols attenuate the inflammatory response of monocytes/macrophages mediated by *F. nucleatum*. We first showed that the tea extracts, EGCG and theaflavins reduce the NF-κB activation induced by *F. nucleatum* in monocytes. Since NF-κB is a key regulator of genes coding for inflammatory mediators, we tested the effects of tea polyphenols on secretion of IL-1β, IL-6, TNF-α, and CXCL8 by macrophages. A pre-treatment of macrophages with the tea extracts, EGCG, or theaflavins prior to a stimulation with *F. nucleatum* significantly inhibited the secretion of all four cytokines and reduced the secretion of MMP-3 and MMP-9, two tissue destructive enzymes. TREM-1 expressed by macrophages is a cell-surface receptor involved in the propagation of the inflammatory response to bacterial challenges. Interestingly, tea polyphenols inhibited the secretion/shedding of soluble TREM-1 induced by a stimulation of macrophages with *F. nucleatum*. The anti-inflammatory properties of tea polyphenols identified in the present study suggested that they may be promising agents for the prevention and/or treatment of periodontal disease and inflammatory bowel disease.

Periodontal disease is defined as a biofilm-associated inflammatory disorder affecting the supporting structures of the teeth, including the periodontal ligament and the alveolar bone. It is one of the most common chronic infections in adults and, if left untreated, may result in tooth loss and systemic complications such as cardiovascular disease, diabetes mellitus, rheumatoid arthritis, preterm birth, and respiratory infections^1^. Periodontal disease results from a dysbiosis in the gingival sulcus that causes a shift from symbiotic host-microbe interactions to a microflora dominated by strictly anaerobic Gram-negative bacterial species^2^. One of these, *Fusobacterium nucleatum*, is known to be present in higher numbers in diseased periodontal sites^3^. Due to its versatile adherence properties, *F. nucleatum* is a key bridging bacterium between early and late colonizers of the subgingival biofilm^4^. *F. nucleatum* is also a common resident of the human gastrointestinal tract. A number of recent reports have indicated that this bacterial species is present in higher numbers in the gastrointestinal tract of patients with inflammatory bowel disease (IBD) and that *F. nucleatum* may contribute to the pathogenesis of colorectal cancer^5^,^6^,^7^.

Inflammation is a complex pathophysiological phenomenon orchestrated by immune cells in response to infections and/or tissue damage. Although inflammation is an integral component of the host defense against pathogens, uncontrolled inflammation caused by a continuous bacterial challenge, as occurs during periodontitis, can lead to extensive secretion by mucosal and immune cells of pro-inflammatory mediators and matrix metalloproteinases (MMPs) that contribute to periodontal tissue destruction^8^. Monocytes and macrophages, which are present in higher numbers in active periodontal lesions than in inactive sites^9^, detect and respond to pathogens via toll-like receptors (TLR) and mediate both inflammation and resolution^10^. Following the recognition of pathogens by the TLR pathway, macrophages secrete a wide variety of cytokines through the nuclear factor-κB (NF-κB) pathway^11^. The Triggering Receptor Expressed on Myeloid Cells 1 (TREM-1) expressed by macrophages is a cell-surface receptor of the immunoglobulin superfamily that is also involved in the propagation of the inflammatory response to bacterial challenges^12^. Soluble TREM-1 (sTREM-1) can be released from the cell surface during the course of an infection and can also be used as a biomarker of systemic inflammation in systemic sepsis^13^, arthritis^14^ and IBD^15^. Recent studies have suggested that the sTREM-1 detected in gingival crevicular fluid, saliva, and serum is a biomarker of periodontal disease and a predictor of therapeutic outcome^16^,^17^,^18^,^19^.

Given the critical role played by the immune/inflammatory response in the pathogenesis of periodontitis that results in the local production of excessive levels of cytokines and tissue destructive enzymes, the modulation of the host response is seen as a valid adjunctive therapy to scaling and root planing in the treatment of periodontitis^20^,^21^. In this regard, plant polyphenols are bioactive molecules of interest due to their anti-inflammatory properties^22^. Tea, an aqueous aromatic infusion of the leaves of the *Camellia sinensis* plant, has a high polyphenol content^23^,^24^. While green tea (non-fermented) contains mainly catechins and their derivatives, the most important being epigallocatechin-3-gallate (EGCG), black tea (fermented) is characterized by the presence of theaflavins and their derivatives^24^,^25^. Many studies have shown that tea polyphenols may contribute to reducing the risk of cardiovascular disease and cancer and can exert a variety of other beneficial effects on human health^24^. In the present study, we investigated the effect of green and black tea polyphenols on *F. nucleatum*-mediated activation of the NF-κB signaling pathway and the secretion of pro-inflammatory mediators and MMPs by monocytes/macrophages.

## Results

### Cell viability assays

An MTT colorimetric assay was used to determine the non-cytotoxic concentrations of the green tea extract, the black tea extract, EGCG, and the theaflavins for use with U937-3xκB and U937 cells. This analysis was required to exclude the possibility that cytotoxicity related to the compounds tested might cause a decrease in NF-κB activation or cytokine and MMP secretion. For the U937-3xκB cell line, the non-cytotoxic concentrations were ≤125 μg/ml for the green tea extract, EGCG, and theaflavins, and ≤500 μg/ml for the black tea extract (Table 1). For the U937 macrophage-like cells, the non-cytotoxic concentrations were ≤62.5 μg/ml for the green tea extract and EGCG, ≤125 μg/ml for the theaflavins, and ≤500 μg/ml for the black tea extract (Table 1).

### NF-κB activation

**Figure 1:**
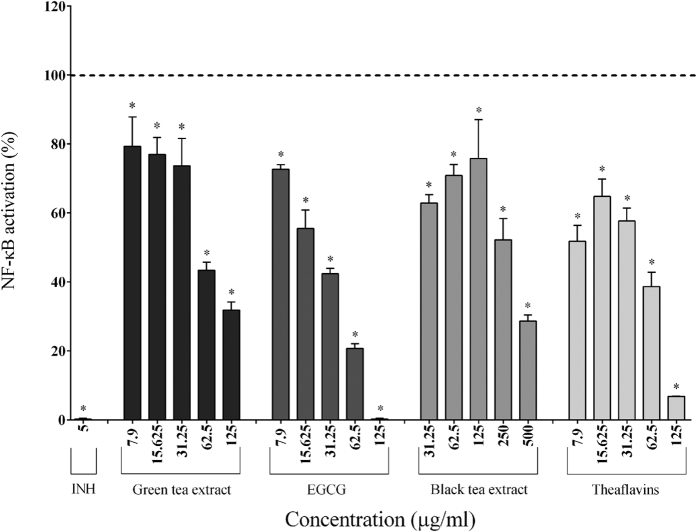
Effect of the green tea extract, EGCG, black tea extract, and theaflavins on *F. nucleatum*-mediated activation of the NF-κB signaling pathway using the U937-3xκB cell model. A value of 100% was assigned to the activation obtained with *F. nucleatum* at an MOI of 100 (-----) in the absence of tea polyphenols. The commercial inhibitor BAY-11-7082 (INH; 5 μg/ml) was used as a positive control. Results are expressed as the means ± SD of triplicate assays from three independent experiments. (*) Significant decrease (*p* < 0.001) compared to untreated *F. nucleatum*-stimulated cells.

The ability of the tea polyphenols to prevent NF-κB activation was evaluated using two-fold serial dilutions beginning at their highest non-cytotoxic concentrations. In the absence of LPS stimulation, the tea polyphenols showed no inhibitory effects on the basal level of NF-κB activity in U937-3xκB cells (data not shown). In general, the tea polyphenols exhibited a comparable dose-dependent inhibitory effect on *F. nucleatum*-induced NF-κB activation in U937-3xκB cells (Fig. 1). More specifically, 62.5 μg/ml of the green tea extract, black tea extract, EGCG, and theaflavins reduced NF-κB activation induced by *F. nucleatum* (MOI of 100) by 56.65%, 29.14%, 79.28%, and 61.37%, respectively while 125 μg/ml of EGCG completely prevented NF-κB activation (Fig. 1). BAY-11-7082 (5 μg/ml), a commercial inhibitor was used as a positive control and totally prevented NF-κB activation.

### Secretion of cytokines, MMPs, and sTREM-1

Since tea polyphenols reduced the activation of the NF-κB signaling pathway, we investigated their effects on inflammatory mediator secretion in a macrophage-like model (PMA-treated U937 cells) stimulated with *F. nucleatum*. Adherent macrophages were pre-treated for 2 h with the tea polyphenols and were then stimulated for 24 h with *F. nucleatum* (MOI of 100). The secretion of cytokines (IL-6, IL-1β, TNF-α, CXCL8) and MMPs (MMP-3, MMP-9) was then measured.

**Figure 2:**
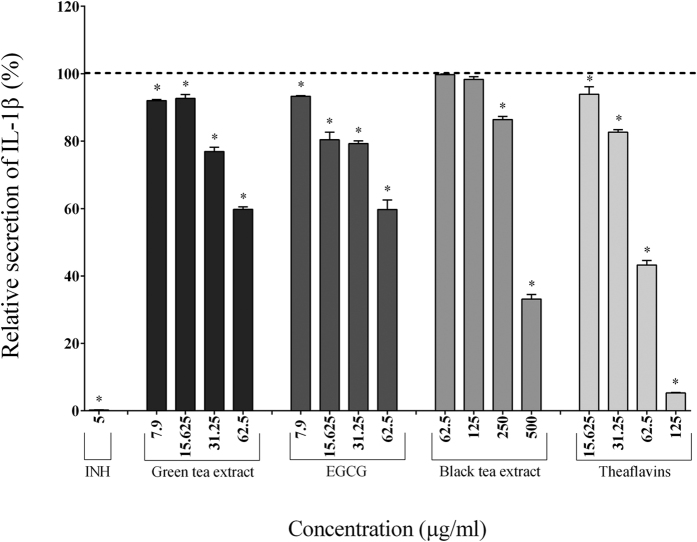
Effect of the green tea extract, EGCG, black tea extract, and theaflavins on the secretion of IL-1β by macrophages stimulated with *F. nucleatum* at an MOI of 100 (-----). The commercial inhibitor BAY-11-7082 (INH; 5 μg/ml) was used as a positive control. Results are expressed as the means ± SD of triplicate assays from three independent experiments. (*) Significant decrease (*p* < 0.001) compared to untreated *F. nucleatum*-stimulated cells.

**Figure 3:**
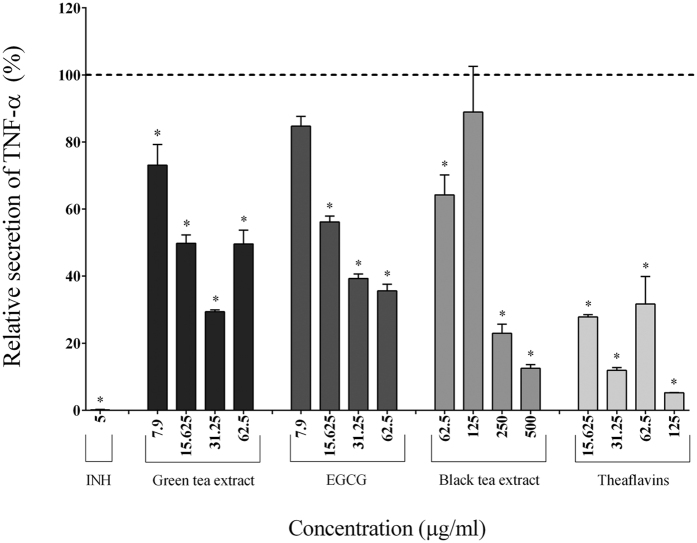
Effect of the green tea extract, EGCG, black tea extract, and theaflavins on the secretion of TNF-α by macrophages stimulated with *F. nucleatum* at an MOI of 100 (-----). The commercial inhibitor BAY-11-7082 (INH; 5 μg/ml) was used as a positive control. Results are expressed as the means ± SD of triplicate assays from three independent experiments. (*) Significant decrease (*p* < 0.001) compared to untreated *F. nucleatum*-stimulated cells.

**Figure 4:**
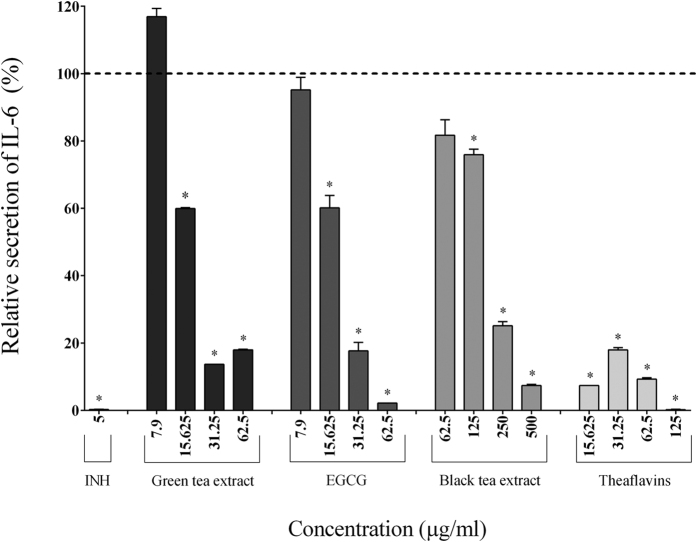
Effect of the green tea extract, EGCG, black tea extract, and theaflavins on the secretion of IL-6 by macrophages stimulated with *F. nucleatum* at an MOI of 100 (-----). The commercial inhibitor BAY-11-7082 (INH; 5 μg/ml) was used as a positive control. Results are expressed as the means ± SD of triplicate assays from three independent experiments. (*) Significant decrease (*p* < 0.001) compared to untreated *F. nucleatum*-stimulated cells.

**Figure 5:**
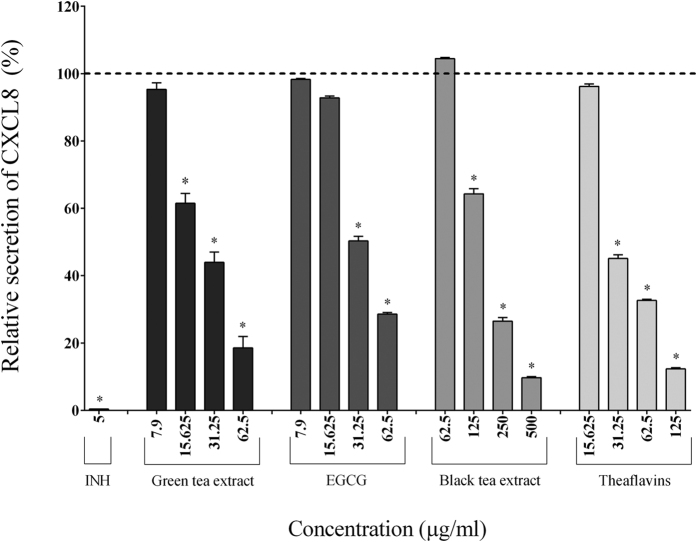
Effect of the green tea extract, EGCG, black tea extract, and theaflavins on the secretion of CXCL8 by macrophages stimulated with *F. nucleatum* at an MOI of 100 (-----). The commercial inhibitor BAY-11-7082 (INH; 5 μg/ml) was used as a positive control. Results are expressed as the means ± SD of triplicate assays from three independent experiments. (*) Significant decrease (*p* < 0.001) compared to untreated *F. nucleatum*-stimulated cells.

**Figure 6:**
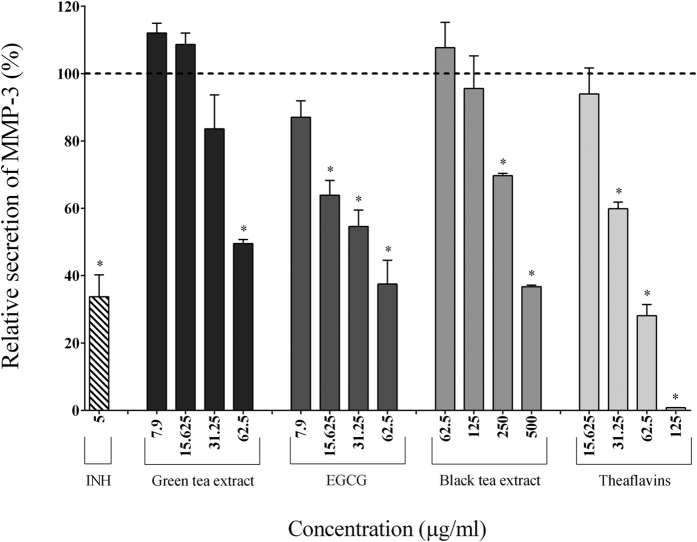
Effect of the green tea extract, EGCG, black tea extract, and theaflavins on the secretion of MMP-3 by macrophages stimulated with *F. nucleatum* at an MOI of 100 (-----). The commercial inhibitor BAY-11-7082 (INH; 5 μg/ml) was used as a positive control. Results are expressed as the means ± SD of triplicate assays from three independent experiments. (*) Significant decrease (*p* < 0.001) compared to untreated *F. nucleatum*-stimulated cells.

**Figure 7:**
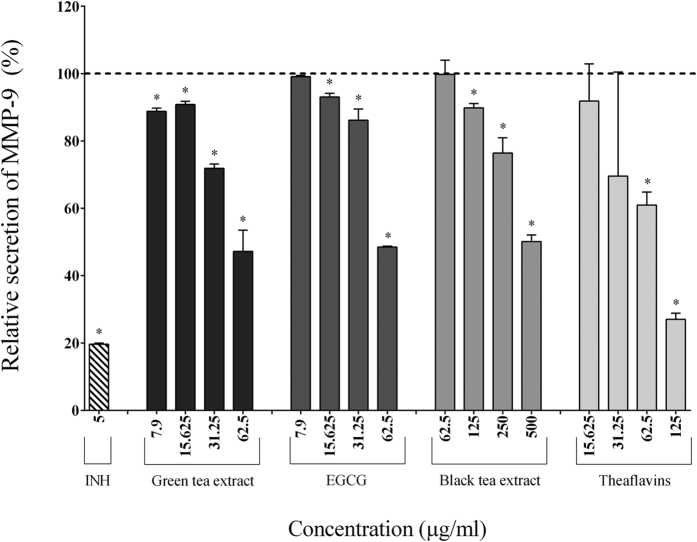
Effect of the green tea extract, EGCG, black tea extract, and theaflavins on the secretion of MMP-9 by macrophages stimulated with *F. nucleatum* at an MOI of 100 (-----). The commercial inhibitor BAY-11-7082 (5 μg/ml) was used as a positive control. Results are expressed as the means ± SD of triplicate assays from three independent experiments. (*) Significant decrease (*p* < 0.001) compared to untreated *F. nucleatum*-stimulated cells.

In the absence of tea polyphenols, the stimulation of macrophages with *F. nucleatum* (MOI of 100) significantly increased the secretion of IL-1β (7.70-fold), TNF-α (84.57-fold), IL-6 (185.25-fold), CXCL8 (32.25-fold), MMP-3 (2.05-fold), and MMP-9 (2.04-fold) (Table 2). The secretion of pro-inflammatory cytokines (IL-1β, IL-6, TNF-α) by macrophages stimulated with *F. nucleatum* was significantly and dose-dependently attenuated by the tea polyphenols compared to the control cells. The green tea extract, EGCG, and theaflavins (62.5 μg/ml) reduced the secretion of IL-1β by 40.49%, 40.27%, and 56.72%, respectively, TNF-α by 50.41%, 64.38%, and 68.30%, respectively, and IL-6 by 82.40%, 97.84%, and 85.39%, respectively (Figs 2, 3 and 4). At 62.5 μg/ml, the black tea extract had weak or no inhibitory effect on cytokine secretion. However, at 250 μg/ml, the black tea extract significantly reduced the secretion of IL-1β (13.57%), TNF-α (77.07%), and IL-6 (74.89%) (Figs 2, 3 and 4). The secretion of CXCL8 by *F. nucleatum*-stimulated macrophages was also significantly inhibited by the green tea extract (≥15.625 μg/ml), black tea extract (≥125 μg/ml), EGCG (≥31.25 μg/ml), and theaflavins (≥31.25 μg/ml) (Fig. 5). Lastly, the secretion of MMP-3 and MMP-9 by *F. nucleatum*-stimulated macrophages was also attenuated by the tea polyphenols. More specifically, at a concentration of 62.5 μg/ml, the green tea extract, EGCG, and theaflavins reduced the secretion of MMP-3 by 50.45%, 62.49%, and 71.91%, respectively, and the secretion of MMP-9 by 52.78%, 51.47%, and 39.02%, respectively (Figs 6 and 7). The black tea extract (250 μg/ml) attenuated the secretion of MMP-3 by 30.32% (Fig. 6) and MMP-9 by 23.59% (Fig. 7).

**Figure 8:**
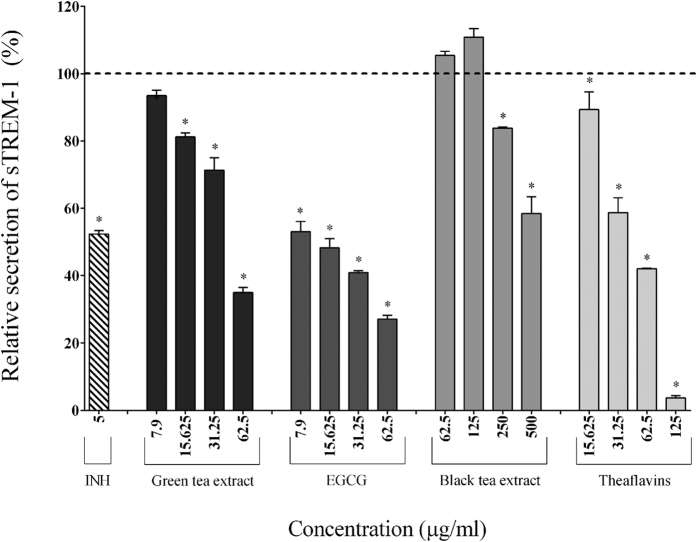
Effect of the green tea extract, EGCG, black tea extract, and theaflavins on the secretion of sTREM-1 by macrophages stimulated with *F. nucleatum* at an MOI of 100 (-----). The commercial inhibitor BAY-11-7082 (5 μg/ml) was used as a positive control. Results are expressed as the means ± SD of triplicate assays from three independent experiments. (*) Significant decrease (*p* < 0.001) compared to untreated *F. nucleatum*-stimulated cells.

We also investigated the ability of tea polyphenols to inhibit the secretion/shedding of sTREM-1 induced by the stimulation of macrophages with *F. nucleatum*. *F. nucleatum* significantly increased the secretion/shedding of sTREM-1 (3.38-fold) (Table 2), while the tea polyphenols caused a significant and dose-dependent inhibition (Fig. 8). More specifically, 62.5 μg/ml of the green tea extract, EGCG, and theaflavins decreased secretion/shedding by 65%, 72.5%, and 57.93%, respectively. The black tea extract (250 μg/ml) reduced the secretion/shedding of sTREM-1 by 16.18%.

## Discussion

*F. nucleatum* is a Gram-negative strictly anaerobic bacterium that is considered a normal resident of the oral cavity. Most research on *F. nucleatum* has focused on its contribution to the pathogenesis of gingivitis and periodontitis through the establishment of a pathogenic subgingival biofilm^4^ and the induction of a host inflammatory response^26^. Recent evidence has shown that *F. nucleatum*, by virtue of its adhesive, invasive, and pro-inflammatory properties, may also be involved in IBD and, consequently, in colorectal cancer^5^,^6^,^7^. This bacterial species colonizes the mucus layer and has been associated with a robust local inflammatory response in human colorectal carcinomas^27^,^28^. Periodontal disease and IBD such as ulcerative colitis and Crohn’s disease share some similarities in terms of their pathogenic process. First, diseased sites contain high numbers of macrophages^29^,^30^. Second, pro-inflammatory cytokines, including TNF-α, IL-1β, and IL-6, are thought to play a critical role in the tissue damage observed with these diseases^31^. Given their anti-inflammatory properties, tea polyphenols show great potential as agents for the prevention and treatment of periodontal disease and IBD. To this end, we investigated the effect of green and black tea polyphenols on the activation of the NF-κB signaling pathway and the secretion of pro-inflammatory mediators by monocytes/macrophages challenged with *F. nucleatum*.

NF-κB is one of the main pathways used by host cells to respond to microbial challenges. Moreover, NF-κB is often referred to as a central mediator of the human immune response because it plays an essential role in several aspects of human health, including the development of innate and adaptive immunity. As such, plant-derived phytochemicals are promising lead compounds for the development of potent and safe inhibitors for cancer and inflammatory disorders driven by NF-κB^32^.

Since tea polyphenols, including EGCG and theaflavins, have been reported to possess immunomodulatory properties^33^, we evaluated the effect of tea polyphenols on *F. nucleatum*-induced NF-κB activation. We previously showed that *F. nucleatum* can strongly activate the NF-κB signaling pathway in the U937-3xκB cell line^34^, as did Kostic *et al*., who suggested that *F. nucleatum* induces an NF-κB-driven pro-inflammatory response that may promote colorectal cancer^35^. We found that 125 μg/ml of EGCG and theaflavins almost completely prevented NF-κB activation. The green tea extract and, to a lesser extent, the black tea extract also attenuated NF-κB activation by *F. nucleatum.* Given that both extracts have a comparable concentration in total polyphenols, it is likely that the differences between the two extracts in regard to inhibition of NF-κB are related to the composition and proportions of individual catechins and theaflavins.

Our results were in agreement with several other studies showing that both green tea catechins and black tea theaflavins inhibit the activation of NF-κB in cultured cell lines. In lipopolysaccharide-activated macrophages and epidermal cells treated with the tumor promoter 12-*O*-tetradecanoylphorbol-13-acetate (TPA), black tea theaflavins and EGCG inhibited the phosphorylation of IκB, preventing NF-κB from translocating to the nucleus and binding to DNA^36^,^37^. Moreover, in intestinal epithelial cells, EGCG is the most potent inhibitor of the IκB kinase activity of intestinal epithelial cells of any green tea catechin^38^. Similarly, the oral administration of EGCG markedly attenuates the severity of acetic acid-induced colitis in rats and reduces TNF-α and IFN-γ levels in plasma as well as NF-κB expression in the colon^39^.

Since NF-κB is a key regulator of over 500 genes, including those coding for the expression of pro-inflammatory mediators^40^, inhibitors of this transcription factor hold great promise for the prevention and treatment of chronic inflammatory disorders^41^. In this context, we investigated the effect of tea polyphenols on inflammatory mediator secretion using a macrophage model stimulated with *F. nucleatum*. Macrophages play a central role in the coordinated resolution of inflammation and the return to tissue homeostasis and are actively involved in all phases of inflammation. They are positioned directly beneath the surface epithelium in the gingiva and the bowel and are the first immune cell population to encounter microbial challenges^29^,^30^. Another important feature of monocytes/macrophages is their ability to secrete cytokines and chemokines. In some cases, this inflammatory response in infected periodontal pockets or in the gastrointestinal tract is continuous, resulting in a chronic inflammatory state. Consequently, the mechanisms underlying the destructive processes associated with periodontitis or IBD are not only related to the direct tissue damage caused by bacteria but also to indirect damage mediated by the uncontrolled host inflammatory response. Most, if not all, of the chronic inflammatory states involved in these pathological conditions are characterized by an overproduction of cytokines (TNF-α, IL-6), chemokines (CXCL8), and MMPs^42^. Elevated levels of these mediators act to amplify the inflammatory process by attracting additional inflammatory cells to the site, thus contributing to tissue destruction and the clinical symptoms observed^42^. In the present study, we showed that green and black tea extracts as well as their major polyphenols, EGCG and theaflavins, significantly and dose-dependently reduce the secretion of three major pro-inflammatory cytokines (IL-1β, TNF-α, and IL-6) by *F. nucleatum*-stimulated macrophages.

IL-1β and IL-6 are signature innate cytokines in periodontal disease and have been associated with inflammatory cell migration and osteoclastogenesis^43^,^44^. In IBD, IL-1β is involved in the increased recruitment of neutrophils and the activation of innate lymphoid cells (ILCs)^45^, while IL-6 activates T cells and macrophages, recruits immune cells, and activates acute-phase proteins. Interestingly, the blockade of IL-1β or IL-6 signaling with monoclonal antibodies is effective in suppressing chronic intestinal inflammation in mouse models. All these findings suggest that these two cytokines may be potential therapeutic targets for the treatment of IBD^45^.

TNF-α is a multi-effect cytokine with many functions, ranging from cell migration to tissue destruction^26^,^45^. Its role and contribution to tissue destruction and bone loss has been clearly documented in periodontal disease^46^. In IBD, TNF-α is a central pro-inflammatory cytokine and causes barrier alterations and promotes the cell death of intestinal epithelial cells and Paneth cells^47^,^48^. TNF-α also promotes tissue destruction by increasing the production of MMPs by myofibroblasts^49^. Interestingly, the administration of an anti-TNF-α monoclonal antibody (infliximab) significantly reduces the clinical symptoms and radiographic and clinical progression of bone loss in both rheumatoid arthritis (human model) and periodontal disease (primate model)^50^,^51^. Moreover, TNF-α-specific antibodies (infliximab and certolizumab) may alleviate IBD by simultaneously suppressing several pro-inflammatory pathways^45^.

*F. nucleatum*-stimulated macrophages secreted higher levels of the chemokine CXCL8 than of any of the other cytokines investigated. Chemokines are chemotactic cytokines that play a very important role in the migration of phagocytic cells to sites of infection^52^,^53^. In addition, CXCL8-mediated chemotactic and activation effects on neutrophils in the inflamed gingiva may contribute to periodontal tissue destruction^54^. The fact that tea polyphenols inhibited the secretion of CXCL8 by macrophages suggested that they have the potential to reduce the influx of inflammatory cells to diseased sites and the amplification of bacteria-induced inflammatory processes. In the case of periodontal disease and IBD, the significant inhibition of the secretion of all the cytokines tested may have a major indirect impact given that anti-inflammatory modalities can indirectly exert antimicrobial effects. Indeed, periodontal or intestinal dysbiosis is crucially dependent on an inflammatory environment since, for example, inflammatory tissue breakdown products are used as nutrients^8^,^55^.

The activation of TREM-1 expressed on neutrophils and monocytes amplifies the expression of various pro-inflammatory cytokines, chemokines, and cell surface receptors^56^. Controlling this amplified inflammatory response may have a significant impact on the severity of periodontal disease and IBD^57^,^58^. Moreover, bacterial challenges induce the release of the soluble form of TREM-1 (sTREM-1) in humans and in mouse models^59^,^60^. Patients with moderate or severe ulcerative colitis and Crohn’s disease have been reported to have increased levels of sTREM-1^61^. sTREM-1 has also been detected in gingival crevicular fluid and is present in higher concentrations in diseased periodontal sites^16^. However, the exact role of sTREM-1 in the inflammatory cascade remains unclear. Some studies have shown that the upregulation of sTREM-1 production is mediated by bacterial challenges^62^ and concluded that it may be a specific marker of infections in various pathologies^13^,^62^. When sTREM-1 is detected in serum it may have been released by circulating leukocytes during the course of a systemic infection or may have been in focal infections, eventually entering the blood stream^63^. Interestingly, we found that tea polyphenols significantly reduce sTREM-1 levels. To the best of our knowledge, no studies have investigated the potential of *F. nucleatum* to induce the secretion/shedding of sTREM-1 or the potential of polyphenols to reduce this trend. Bostanci *et al*. recently showed that *P. gingivalis* induces TREM-1 expression in monocytes concomitantly with an increased release of sTREM-1^64^. They also reported that doxycycline reduces the expression of TREM-1 and the secretion of sTREM-1^65^.

Macrophages produce several MMPs, which are a group of genetically distinct but structurally-related enzymes involved in the degradation of extracellular matrix and basement membrane proteins during normal tissue turnover and tissue destructive processes^66^. MMP-3 and MMP-9 have been strongly associated with the progression of periodontitis^66^. In IBD, particularly Crohn’s disease, MMPs such as collagenases and stromelysins, which can degrade the extracellular matrix, cause ulceration and result in tissue destruction^67^,^68^. High levels of extracellular MMPs, which can be upregulated by pro-inflammatory cytokines, have been detected in areas of tissue injury and in ulceration foci in IBD patients^68^. MMP inhibitors efficiently prevent tissue destruction in other inflammatory processes such rheumatoid arthritis, aphthous disease of oral mucosa, and periodontal disease^69^,^70^. Interestingly, we showed that tea polyphenols, including EGCG and theaflavins, reduce MMP-3 and MMP-9 secretion by *F. nucleatum*-stimulated macrophages. These findings were in agreement with those of a study by Oka *et al*., who showed that MMP-2 and MMP-9 activities are lower in theaflavin-treated rat osteoclast precursor cells than in control osteoclasts^71^ and of a study by Yun *et al*., who reported that EGCG has an inhibitory effect on the gene expression of MMP-9 in osteoblasts and on the formation of osteoclasts^72^.

## Conclusion

In conclusion, we investigated the anti-inflammatory potential of green and black tea extracts, EGCG, and theaflavins using *in vitro* models related to periodontal disease and IBD. All tea compounds tested exhibited comparable effects, including the capacity to reduce the activation of NF-κB, the secretion of pro-inflammatory mediators and MMPs, and the secretion/shedding of sTREM-1. Given that pathological inflammation involves a loss of tolerance and/or of regulatory processes, the anti-inflammatory properties of tea polyphenols suggested that they may represent promising preventive or therapeutic agents.

## Materials and Methods

### Tea polyphenols

The commercial green and black tea extracts (Hangzhou Gosun Biotechnologies, China) had polyphenol contents of 98.4% and 92%, respectively. Stock solutions were freshly prepared by dissolving the tea powder (20 mg of green tea extract or 10 mg of black tea extract) in 1 ml of sterile warm distilled water and filtering the solution through a 0.22-μm pore size membrane filter. EGCG (Sigma-Aldrich, Canada), the predominant catechin in the green tea extract (47.9%), was also dissolved in sterile distilled water at a concentration of 10 mg/ml and was sterilized by filtration. The theaflavin preparation was purchased from DeHe Biotechnology (China). According to the product specifications, the preparation is a mixture of theaflavin, theaflavin-3-gallate, theaflavin-3′-gallate, and theaflavin-3,3′-digallate, with more than 80% purity. A stock solution was prepared by dissolving 20 mg of powder in 1 ml of 95% ethanol.

### Bacteria and growth conditions

*F. nucleatum* ATCC 25586 was grown anaerobically (80% N_2_, 10% CO_2_, 10% H_2_) for 24 h at 37 °C in Todd-Hewitt broth (THB; Becton, Dickinson and Company, USA) supplemented with 0.001% hemin and 0.0001% vitamin K.

### Activation of the NF-κB transcription factor

The human monoblastic leukemia cell line U937 3xκB-LUC, a subclone of the U937 cell line stably transfected with a luciferase gene coupled to a promoter of three NF-κB- binding sites, was kindly provided by R. Blomhoff (University of Oslo, Norway)^73^. The cells were routinely cultivated in Roswell Park Memorial Institute 1640 medium (RPMI-1640; Life Technologies Inc., Canada) supplemented with 10% heat-inactivated fetal bovine serum (FBS), 100 μg/ml of penicillin G/streptomycin, and 75 μg/ml of hygromycin B at 37 °C in a 5% CO_2_ atmosphere. To investigate the effect of tea polyphenols on *F. nucleatum*-induced NF-κB activation, U937 3xκB-LUC cells (10^6^ cells/ml) were pre-incubated with the compounds (non-cytotoxic concentrations; in RPMI containing 1% FBS) for 30 min in the wells of a black wall, black bottom 96-well microplate (Greiner Bio-One North America Inc., USA). They were then stimulated for 6 h with *F. nucleatum* at a multiplicity of infection (MOI) of 100. The bacterial suspension was prepared from an overnight culture of *F. nucleatum*. Wells with no *F. nucleatum* or no compounds were used as controls. An assay using the commercial inhibitor BAY-11-7082 (5 μg/ml; EMD Millipore, Canada) was used as a positive control for the inhibition of the NF-κB signaling pathway. NF-κB activation was determined by measuring luciferase activity following the addition of Bright-Glo reagent (Promega Corporation, USA) in accordance with the manufacturer’s protocol. Luminescence was monitored using a Synergy 2 microplate reader (BioTek Instruments, USA).

### Cytokine and MMP secretion by macrophages

U937 human monocytes (CRL-1593.2; American Type Culture Collection, USA) were cultivated in RPMI-1640 supplemented with 10% heat-inactivated FBS and 100 μg/ml of penicillin G/streptomycin at 37 °C in a 5% CO_2_ atmosphere. The monocytes (2.5 × 10^5^ cells/ml) were incubated in RPMI-10% FBS containing 100 ng/ml of phorbol myristic acid (PMA; Sigma-Aldrich, Canada) for 48 h to induce differentiation into adherent macrophage-like cells^74^. The adherent macrophage-like cells were harvested by gentle scraping followed by centrifugation at 1,200 × g for 5 min. The cells were washed, suspended in RPMI-1% FBS at a concentration of 1 × 10^6^ cells/ml, seeded in the wells of a 12-well microplate (1 × 10^6^ cells/well), and incubated overnight at 37 °C in a 5% CO_2_ atmosphere. The macrophage-like cells were then pre-treated for 2 h with either green tea extract, black tea extract, EGCG, or theaflavins (non-cytotoxic concentrations; in RPMI containing 1% FBS) prior to being stimulated with *F. nucleatum* at an MOI of 100. An assay using a commercial inhibitor (BAY-11-7082; 5 μg/ml) was used as a positive inhibitory control. After a 24-h incubation at 37 °C in a 5% CO_2_ atmosphere, the culture medium supernatants were collected and were stored at −20 °C until used. Cells incubated in culture medium with or without tea polyphenols and stimulated or not with bacteria were used as controls. Enzyme-linked immunosorbent assay (ELISA) kits (eBioscience Inc., USA; R&D Systems, USA) were used to determine IL-1β, IL-6, CXCL8, TNF-α, MMP-3, MMP-8, MMP-9, and sTREM-1 concentrations according to the manufacturers’ protocols.

### Effects of tea polyphenols on cell viability

Prior to evaluating the anti-inflammatory properties of tea polyphenols, we determined their cytotoxic effect on the two cell lines to exclude the possibility that toxicity related to the compounds might cause a decrease in NF-κB activation or in cytokine and MMP secretion. The cells were treated for 24 h with either green tea extract, black tea extract, EGCG, or theaflavins at concentrations of 500, 250, 125, 62.5, 31.25, 15.625, and 7.9 μg/ml. A colorimetric MTT cell viability assay (Roche Diagnostics, Germany) using 3-[4,5-diethylthiazol-2-yl]-2,5-diphenyltetrazolium bromide as the substrate was performed according to the manufacturer’s protocol. Untreated control cells were assigned a value of 100%.

### Statistical analysis

Unless indicated otherwise, all experiments were performed in triplicate in three independent experiments. The data are expressed as means ± standard deviations (SD). Statistical analyses were performed using a one-way analysis of variance with a post hoc Bonferroni multiple comparison test (GraphPad Software Inc., USA). All results were considered statistically significant at *p* < 0.001.

## Additional Information

**How to cite this article**: Lagha, A. B. and Grenier, D. Tea polyphenols inhibit the activation of NF-κB and the secretion of cytokines and matrix metalloproteinases by macrophages stimulated with *Fusobacterium nucleatum*. *Sci. Rep.* **6**, 34520; doi: 10.1038/srep34520 (2016).

## Figures and Tables

**Table 1:** Effect of the green tea extract, EGCG, black tea extract, and theaflavins on the viability of U937-3xκB and U937 macrophage-like cells.

Compounds	Cell viability (%)	
	U937-3xκB	U937 macrophage-like cells
Green tea extract (μg/ml)		
250	83.3 ± 6.7	58.4 ± 8.2
125	99.0 ± 7.3	89.2 ± 6.6
62.5	95.7 ± 1.8	113.4 ± 14.2
EGCG (μg/ml)		
250	74.7 ± 3.5	17.5 ± 18.7
125	95.2 ± 2.5	46.6 ± 15.7
62.5	99.8 ± 5.2	87.5 ± 3.1
Black tea extract (μg/ml)		
500	94.0 ± 2.6	98.7 ± 8.1
250	91.9 ± 3.6	101.7 ± 8.5
125	95.24 ± 1.3	107.2 ± 5.2
Theaflavins (μg/ml)		
500	35.2 ± 6.0	41.0 ± 3.0
250	72.9 ± 2.7	67.6 ± 4.6
125	96.8 ± 8.2	108.8 ± 11.0
None	100 ± 0.8	100 ± 2.3

**Table 2:** Secretion of IL-1β, TNF-α, IL-6, CXCL8, MMP-3, MMP-9, and sTREM-1 by *F. nucleatum*-stimulated macrophages.

Inflammatory mediators	Concentration (pg/ml)		Fold increase
	Control	*F. nucleatum* (MOI = 100)	
IL-1β	33.87 ± 1.67	260.9 ± 0.41	7.70
TNF-α	41.08 ± 29.05	3474 ± 26.25	84.57
IL-6	3.180 ± 0.54	589.1 ± 22.27	185.25
CXCL8	11359 ± 3142	366371 ± 1914	32.25
MMP-3	19676 ± 68.69	40295 ± 3723	2.05
MMP-9	118651 ± 1063	242000 ± 2465	2.04
sTREM-1	685.5 ± 1.98	2317 ± 66.10	3.38
